# Supplementary material for: Deoxynivalenol and Zearalenone: Different Mycotoxins with Different Toxic Effects in the Sertoli Cells of Equus asinus
Source: Cells. 2021 Jul 27;10(8):1898. doi: 10.3390/cells10081898 (PMC8394322; doi:10.3390/cells10081898)
Supplement: Supplementary file 1 [file cells-10-01898-s001.zip › cells-1286064-supplmementary.pdf]

Figure S1

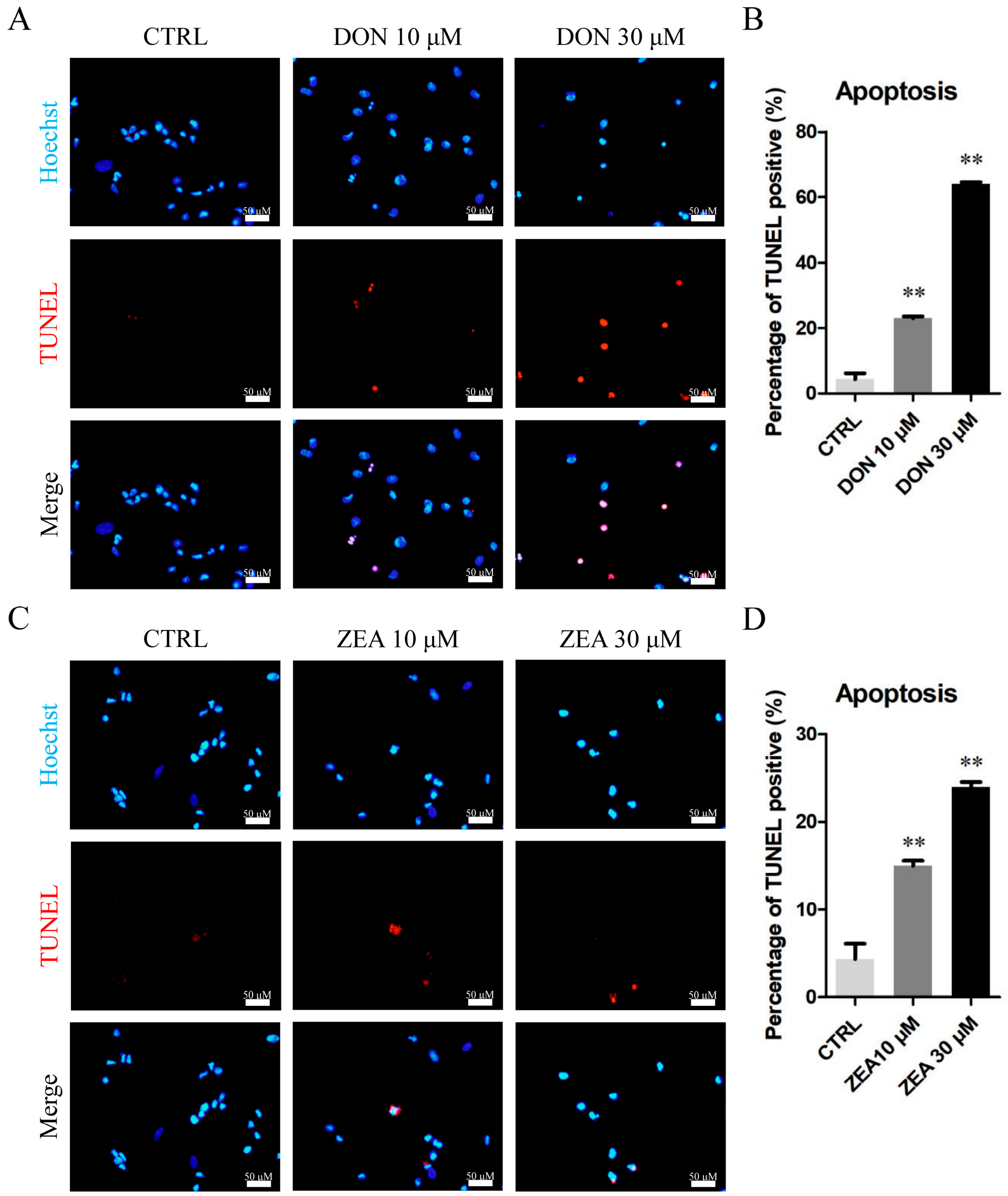

Figure S1. DON and ZEA exposure increasing apoptosis rate in cultured SCs. (Title) SCs were stained blue with Hoechst33342 solution. TUNEL assay was performed using immunostaining. (**A**) Immunofluorescent staining of TUNEL and Hoechst33342 of DON treated SCs. Bar indicates 50 μm. (**B**) The percentages of TUNEL positive SCs exposed to DON. (**C**) Immunofluorescent staining of TUNEL and Hoechst33342 of ZEA treated SCs. Bar indicates 50 μm. (**D**) The percentages of TUNEL positive SCs exposed to ZEA. The results are presented as mean ± SD. All experiments were repeated at least three times. $p < 0.05$; * $p < 0.01$.

Figure S2

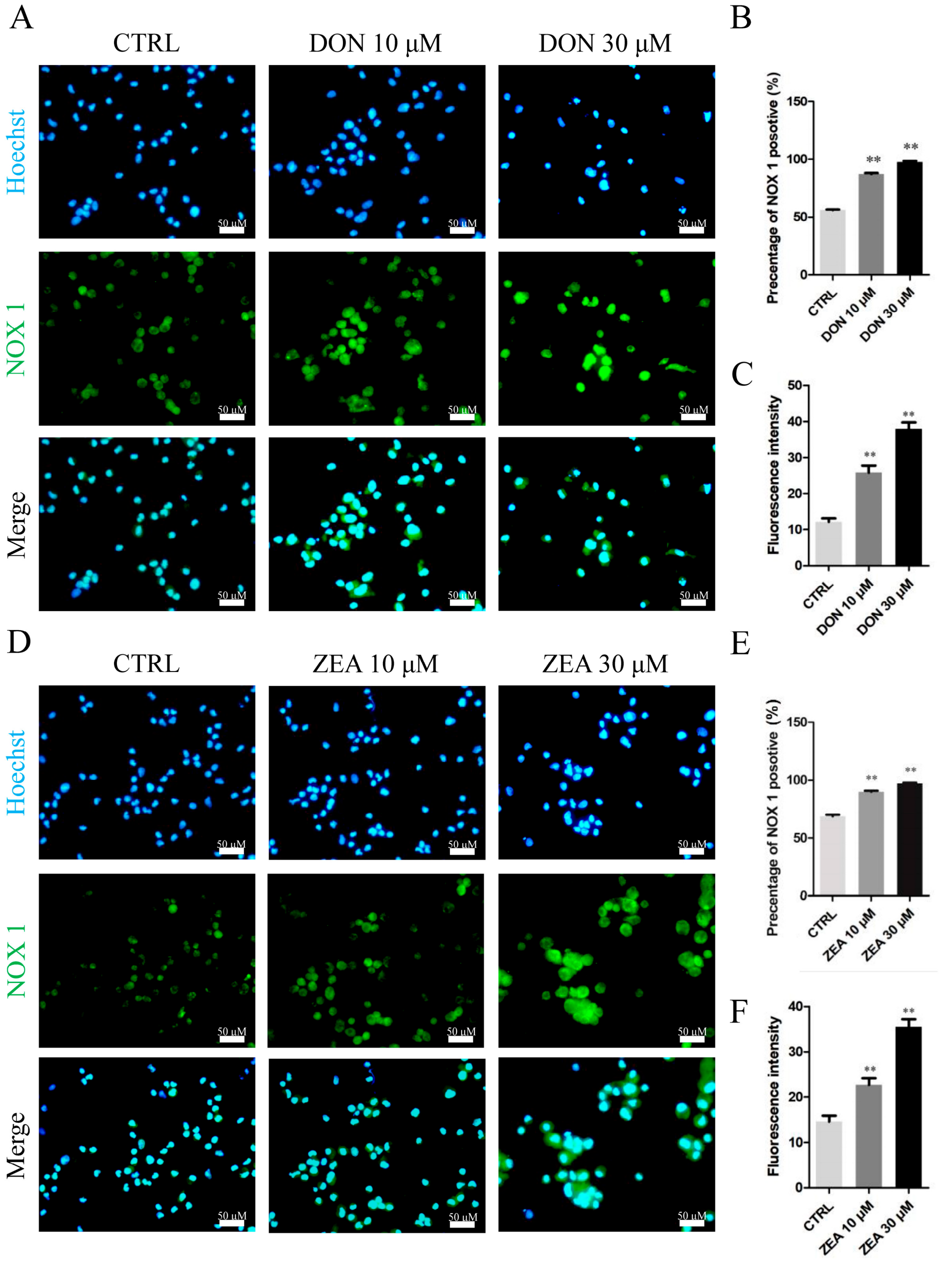

Figure S2. Immunofluorescence assay probing the expression of SCs phosphor- NOX1 proteins in DON (**A**) and ZEA (**D**) treatment groups. (Title) The percentages of positive cells (**B**/**E**) and fluorescence intensity (**C**/**F**) were analyzed respectively. Bar indicates 50 μm. Data are presented as means ± SD. $p < 0.05$; * $p < 0.01$.

Figure S3

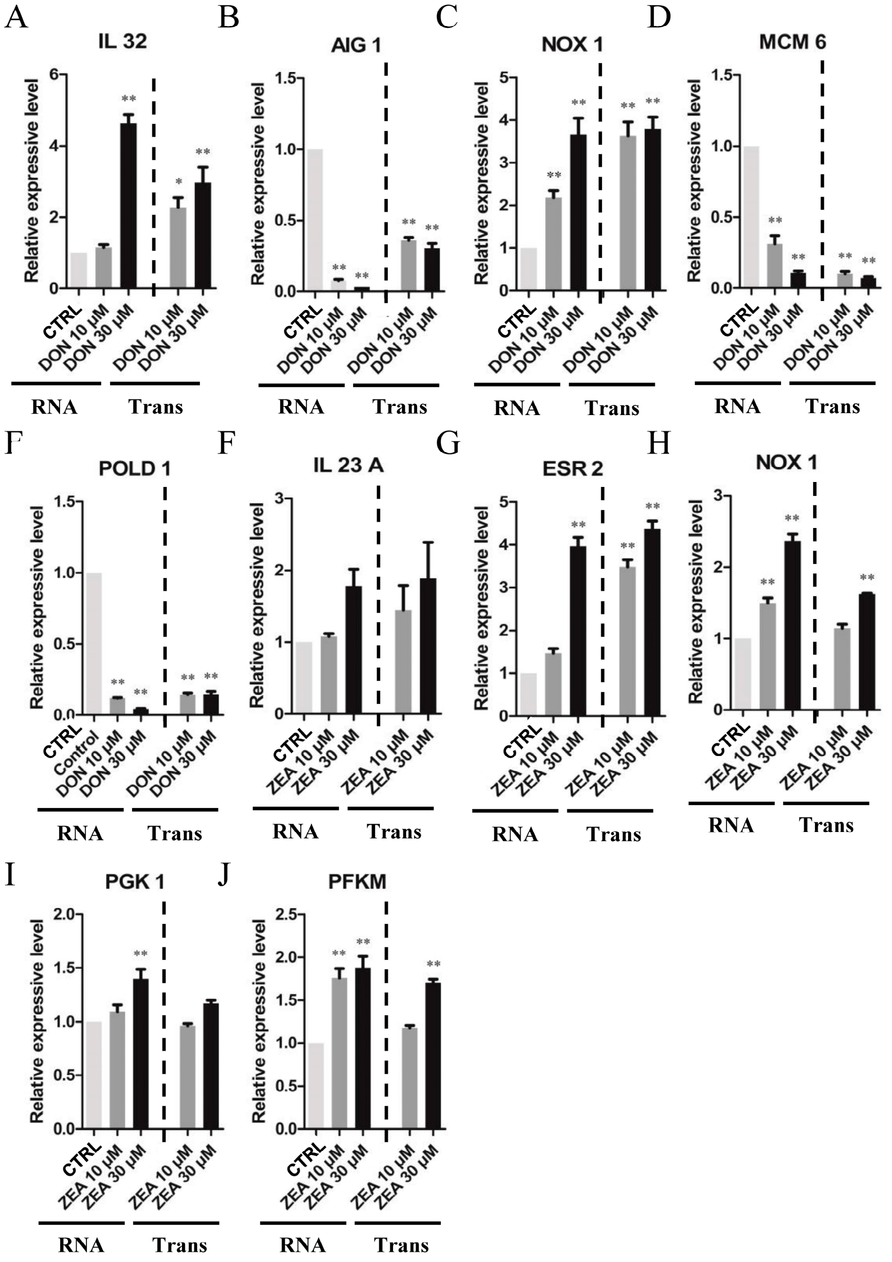

Figure S3. DON and ZEA exposure affecting mRNA abundance of tumorigenesis related genes in cultured GCs. (Title) (**A–E**) Quantitative RT-PCR for IL32, AIG1, NOX1, MCM6, and POLD1 in DON treatment groups. (**F–J**) IL32A, ESR2, NOX1, PGK1, and PFKM in ZEA exposure groups. The mRNA levels of all genes were normalized to SCs GAPDH gene. The results are presented as mean ± SD. All experiments were repeated at least three times. $p < 0.05$; * $p < 0.01$.
